# Lightweight Language Models are Prone to Reasoning Errors for Complex Computational Phenotyping Tasks

**Published:** 2025-07-30

**Authors:** Shashank Yadav, David Maughan, Vignesh Subbian

**Affiliations:** College of Engineering, The University of Arizona, Tucson, AZ; College of Engineering, The University of Arizona, Tucson, AZ; College of Engineering, The University of Arizona, Tucson, AZ

**Keywords:** Large Language Models, Computer Reasoning, Generative Artificial Intelligence, Computational Phenotyping, Electronic Phenotyping

## Abstract

**Objective:**

Although computational phenotyping is a central informatics activity with resulting cohorts supporting a wide variety of applications, it is time-intensive because of manual data review. We previously assessed the ability of LLMs to perform computational phenotyping tasks using computable phenotypes for ARF respiratory support therapies. They successfully performed concept classification and classification of single-therapy phenotypes, but underperformed on multiple-therapy phenotypes. To understand issues with these complex tasks, we expanded PHEONA, a generalizable framework for evaluation of LLMs, to include methods specifically for evaluating faulty reasoning.

**Materials and Methods:**

We assessed the responses of three lightweight LLMs (DeepSeek-r1 32 billion, Mistral Small 24 billion, and Phi-4 14 billion) both with and without prompt modifications to identify explanation correctness and unfaithfulness errors for phenotyping.

**Results:**

For experiments without prompt modifications, both errors were present across all models although more responses had explanation correctness errors than unfaithfulness errors. For experiments assessing accuracy impact after prompt modifications, DeepSeek, a reasoning model, had the smallest overall accuracy impact when compared to Mistral and Phi.

**Discussion:**

Since reasoning errors were ubiquitous across models, our enhancement of PHEONA to include a component for assessing faulty reasoning provides critical support for LLM evaluation and evidence for reasoning errors for complex tasks. While insights from reasoning errors can help prompt refinement, a deeper understanding of why LLM reasoning errors occur will likely require further development and refinement of interpretability methods.

**Conclusion:**

Reasoning errors were pervasive across LLM responses for computational phenotyping, a complex reasoning task.

## BACKGROUND AND SIGNIFICANCE

Computational phenotyping is a central informatics activity that has supported a variety of key downstream tasks, including recruitment for clinical trials, development of clinical decision support systems, and hospital quality reporting.[1–4] The development of computable phenotypes traditionally involves identifying and constructing relevant data elements for classification and applying an algorithm to generate the desired cohort(s).[5] However, these methods are often time and resource-intensive due to the need for manual review of these elements.[3] To advance development of next-generation computational phenotyping methods, we assessed the ability of lightweight Large Language Models (LLMs) to phenotype encounters using computable phenotypes for Acute Respiratory Failure (ARF) respiratory support therapies. We found that several lightweight LLMs performed well for single-therapy phenotypes, but they underperformed on multi-therapy phenotypes. Thus, a natural next step is to explore why these performance deficits occurred to better adapt lightweight LLMs for computational phenotyping tasks to reduce the overall need for time-intensive manual review.

One method for troubleshooting performance issues with LLMs is reviewing Chain-of-Thought (CoT) responses to evaluate each model’s thought processes and identify reasoning errors.[6–11] With CoT, which was the prompt engineering technique we employed for our computational phenotyping tasks,[4] the model constructs its final response through a series of intermediate reasoning steps.[12] While we previously identified and demonstrated methods for assessing factual errors by evaluating answer correctness and hallucination frequency and severity,[4] there remain several opportunities for expanding these methods to include assessment of faulty reasoning in LLM-based applications to computational phenotyping tasks. Specifically, we can assess the presence of logical inconsistencies, or issues with explanation correctness,[6] and we can assess the presence of unfaithfulness.[13] Evaluation of explanation correctness can allow better understanding of where illogical reasoning affected overall performance. Meanwhile, recent studies have indicated that CoT responses are frequently unfaithful to the internal reasoning process of the model.[13–16] Since unfaithfulness can potentially affect model accuracy and undermine the analysis of faulty reasoning in LLM responses, a deeper understanding of when and how frequently unfaithfulness occurs is critical to an accurate view of LLM reasoning.

While many recent studies have assessed the ability of LLMs to reason, only a handful have assessed reasoning errors. Additionally, most of these studies have assessed unfaithfulness[13–17] with only one[6] explicitly evaluating explanation correctness. Previous studies exploring unfaithfulness included experiments both with[13, 15, 17] and without[14] introducing biases, nudges, or reasoning errors in the prompt. However, these studies leave several gaps in unfaithfulness research. First, there are deficits in understanding unfaithfulness specifically for complex reasoning tasks, such as computational phenotyping. Of the studies exploring unfaithfulness, only one provided results for a complex reasoning task: answering short-answer questions from the Putnam mathematics exam.[14] Furthermore, few unfaithfulness studies have been performed with lightweight LLMs and none have explored unfaithfulness without prompt modifications in lightweight LLMs. Thus, there are opportunities to better understand reasoning errors by extending unfaithfulness studies to include responses from lightweight LLMs, evaluating additional types of reasoning errors, and conducting these experiments within the context of computational phenotyping, a complex reasoning task.

## OBJECTIVE

The primary objective of this study was to answer the following overarching question: How frequently do reasoning errors occur in lightweight LLM responses to complex computational phenotyping tasks and how might these errors impact model performance? In addressing this question, we made the following contributions:

1. We developed experiments for evaluating reasoning errors, specifically explanation correctness and unfaithfulness errors, in LLM responses.
2. We performed a demonstration of these experiments by assessing reasoning errors in responses for a specific computational phenotyping use case.
3. We provided empirical evidence for the presence of reasoning errors in lightweight LLMs for complex tasks, specifically computational phenotyping.

## MATERIALS AND METHODS

We first describe the experiments designed to evaluate faulty reasoning in LLM-based applications for computational phenotyping. We then discuss how these experiments were conducted for phenotyping encounters to assess the presence of reasoning errors in model responses.

### Evaluation of Reasoning Errors

**Figure 1:**
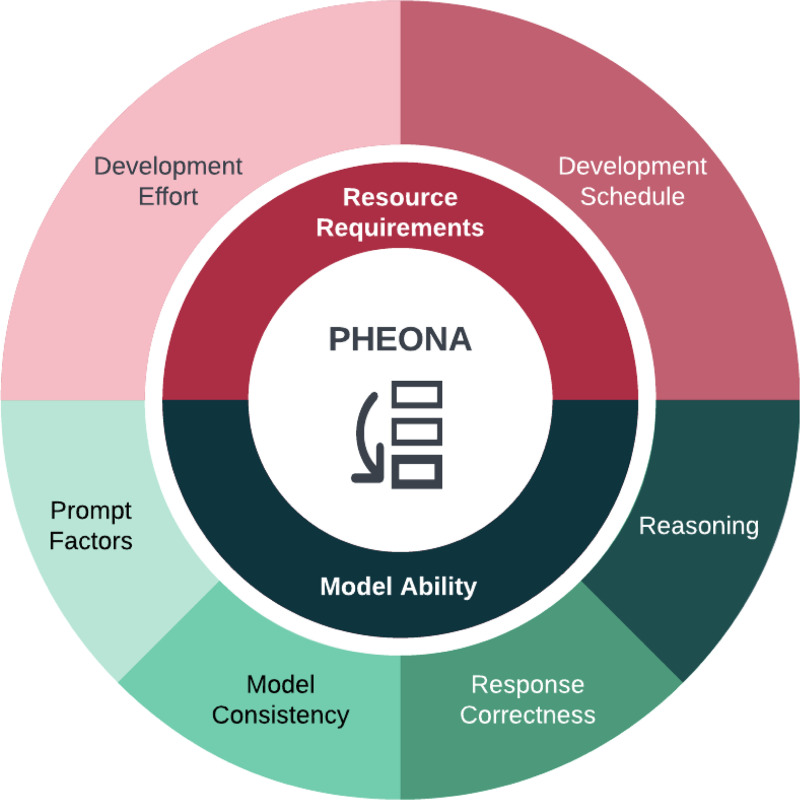
Overview of the components and related criteria of PHEONA (Evaluation of PHEnotyping for Observational Health Data) with an updated component, *Reasoning*, for evaluating faulty reasoning in model responses.

We previously developed PHEONA (Evaluation of PHEnotyping for Observational Health Data), an evaluation framework for LLM-based applications to computational phenotyping.[18] In this study, we expanded PHEONA to include *Reasoning*, a component for evaluating faulty reasoning in CoT responses. We based the criteria for this component on a previously developed framework for evaluating CoT reasoning.[6] In particular, we adapted the metrics of *Explanation Correctness Errors*, which assesses the model’s ability to produce logically sound reasoning, and *Explanation Completeness*, which evaluates the model’s ability to include all necessary reasoning components in its response. We kept *Explanation Correctness Errors* as-is and then considered *Unfaithfulness* instead of *Explanation Completeness* since *Unfaithfulness* is a more widely recognized concept. Therefore, we were able to test logical inconsistencies and unfaithful reasoning in the CoT responses. Additionally, within the *Unfaithfulness* component, we outlined experiments for unfaithfulness based on previously conducted experiments both with[13, 15, 17] and without[14] prompt modifications. Specifically, the experiments without prompt modifications were the *Restoration Errors* and *Unfaithful Shortcut Errors* experiments while the experiments with prompt modifications were the *Few-Shot Biasing* and *Hint Biasing* experiments. The updated PHEONA framework and details of both criteria are presented in Figure 1 and Table 1.

### Phenotyping Use Case

We used previously developed computable phenotypes for Acute Respiratory Failure (ARF) respiratory support therapies.[19] Encounters were classified based on the type and order of respiratory support therapies received. The eight phenotypes of interest were 1) Invasive Mechanical Ventilation (IMV) only; 2) Noninvasive Positive Pressure Ventilation (NIPPV) only; 3) High-Flow Nasal Insufflation (HFNI) only; 4) NIPPV Failure (or NIPPV to IMV); 5) HFNI Failure (or HFNI to IMV); 6) IMV to NIPPV; 7) IMV to HFNI; and 8) None. We previously applied LLMs to the task of phenotyping encounters using these phenotypes.[4] In this study, we revisited this process to apply our reasoning experiments since it is a complex reasoning task with varying levels of model performance.

### Data Source and Processing

We used the eICU Collaborative Research Database (eICU-CRD) database since it was used for development of the original phenotypes.[19, 20] The eICU-CRD database contains timestamped Intensive Care Unit (ICU) data from the Philips Healthcare eICU program for continuous monitoring of intensive care patients.[20] We constructed the descriptions for each encounter by concatenating all of the concepts within each encounter that were relevant to the respiratory therapies and medications of interest after classifying the concepts using LLMs.[4, 18] Each individual concept was formatted as *“#:* {*concept*}*”*, where “#” represented the order of the concept in the encounter records based on its first occurrence.

### Model Selection

We selected LLM models available at the time of this study from Ollama, an open-source package that establishes local connections with open-source LLM models.[21] Due to graphical processing unit (GPU) availability, we selected the following lightweight, instruction-tuned models for testing: Mistral Small 24 billion with Q8.0 quantization (model tag: 20ffe5db0161; identified as Mistral), Phi-4 14 billion with Q8.0 quantization (model tag: 310d366232f4; identified as Phi), and DeepSeek-r1 32 billion with Q4 K M quantization (model tag: edba8017331d; identified as DeepSeek).[21] Unlike Mistral and Phi, DeepSeek is a reasoning model trained with reinforcement learning to produce a detailed thought-process prior to returning the final answer.[22] Since DeepSeek was not previously used for phenotyping due to high response latencies, we used Mistral’s constructed descriptions as the inputs. All models were executed on a single Nvidia V100 32GB GPU. Temperature and top-p were set at 0.50 and 0.99, respectively.

### Prompt Engineering

We used almost the same base prompts for our experiments as those used previously for phenotyping except we modified the instructions to remove all requests for responses to be returned within a certain number of sentences to ensure full CoT responses (Supplementary Material B). In addition to modifying prompts for the *Few-Shot* and *Hint Biasing* experiments, we also modified the prompts to assess faulty reasoning errors by “levels” of CoT using three prompts: *No CoT*, *Some CoT*, and *Full CoT*. The *Full CoT* prompt was the original prompt used for phenotyping with 8 total questions across 4 broader sections. The *No CoT* prompt did not have any reasoning questions and only asked the LLM to return the final answer while the *Some CoT* prompt included a single question for each of the 4 broader sections within the *Full CoT* prompt to guide the model through some, but not all, of the thought processes required to assign the final phenotype.

### Experiments

The following sections detail the individual experiments we performed to demonstrate the specific criteria within the *Reasoning* component.

#### Explanation Correctness

To assess *Explanation Correctness Errors*, we randomly sampled 100 constructed descriptions for each model and retrieved responses for all CoT prompts without modifying the prompts. We manually reviewed each response for logical inconsistencies. Logical inconsistencies were assessed by first identifying statements in the format of logical reasoning, i.e., *premise*⇒*conclusion*,[6] and then assessing whether the *conclusion* was inconsistent with the *premise*, or known facts, or established rules. For example, the following statement is logically inconsistent because IMV requires intubation: “The patient was not intubated so the final classification is IMV Only.”

#### Unfaithfulness

The *Restoration Errors* and *Unfaithful Shortcuts Errors* were assessed by reviewing CoT responses without making any prompt modifications. After randomly shuffling the entire dataset of constructed descriptions, we generated LLM responses until we had 100 total correct answers across all CoT prompts based on the ground truths from the original phenotyping study.[19] We only wanted responses that identified the correct phenotype to avoid conflating unfaithful reasoning with incorrect reasoning. The *Few-Shot Biasing* and *Hint Biasing* experiments were then performed using prompt modifications. For the *Few-Shot Biasing* experiments, we included an examples section within the prompt (Supplementary Material B). For the random examples, we shuffled the dataset and took the first 3 constructed descriptions for each model. For the specific examples, we identified the phenotype with the least number of instances in the phenotyping ground truth and identified 3 random constructed descriptions for this phenotype for each model. For the *Hint Biasing* experiments, we added a hint within the prompt of the following structure: *I think that the answer is <ground truth>. If you use this information, please indicate this in your response*, where *<ground truth>* was replaced with the subsequent phenotype based on the order outlined in the use case. For example, if the ground truth was IMV Only, the subsequent ground truth was NIPPV Only and if the ground truth was None, the subsequent ground truth was IMV Only. For these experiments, we tested all possible combinations of few-shot and hinting. We made the corresponding prompt modifications, shuffled the dataset, and then retrieved the first 1,000 responses for the base prompts and for each experimental combination. Since we were interested in determining when the biased responses differed from the unbiased responses, we selected all responses, even if they were not correct based on the ground truths. However, we did not select any responses where the actual ground truth was the phenotype with the least number of instances to better distinguish between correct and unfaithful reasoning.

### Evaluation

For the *Explanation Correctness Errors*, *Restoration Errors*, and *Unfaithful Shortcut Errors* experiments, when reviewing the responses, if there was at least one instance of the error within the response, it was considered a positive instance of the error. The positive instances were summed across each model and CoT type. However, due to differences between the models, there were slight differences in how each model response was assessed. Since DeepSeek is a reasoning model, it responds with a *think* section prior to all of the specific CoT questions while the remaining models only provide this reasoning for the specific CoT questions in the prompt. Therefore, when reviewing model responses, we evaluated the CoT responses for Mistral and Phi and the *think* section for DeepSeek. Furthermore, since the *No CoT* section only produced the selected phenotype for Mistral and Phi, we only included results for *No CoT* when we were assessing accuracy rather than response content. Meanwhile, for the *Few-Show Biasing* and *Hint-Biasing* experiments, we calculated the accuracy for each experimental combination to assess performance impacts of each bias and thus, we included results for the *No CoT* prompts for all tested models.

## RESULTS

### Phenotyping Use Case

There were 159,701 encounters and patients, after limiting to the first encounter for each patient,[4]. Using the previously developed phenotyping algorithm, the encounters were phenotyped as follows: 16,736 (10.5%) as IMV only; 6,833 (4.3%) as NIPPV only; 1,089 (0.7%) as HFNI only; 1,466 (0.9%) as NIPPV Failure; 568 (0.4%) as HFNI Failure; 601 (0.4%) as IMV to NIPPV; 186 (0.1%) as IMV to HFNI; and 132,222 (82.8%) as None.[19] Since IMV to HFNI had the least number of instances, it was used for the examples in the specific few-shot prompts.

### Experimental Results

**Figure 2:**
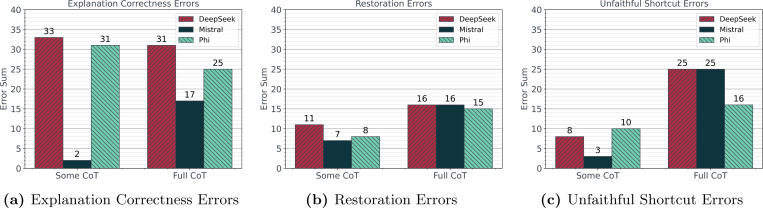
Total number of responses with explanation correctness, restoration, and unfaithful shortcut errors across 100 phenotyped constructed descriptions for each model and Chain-of-Thought (CoT) type.

**Figure 3:**
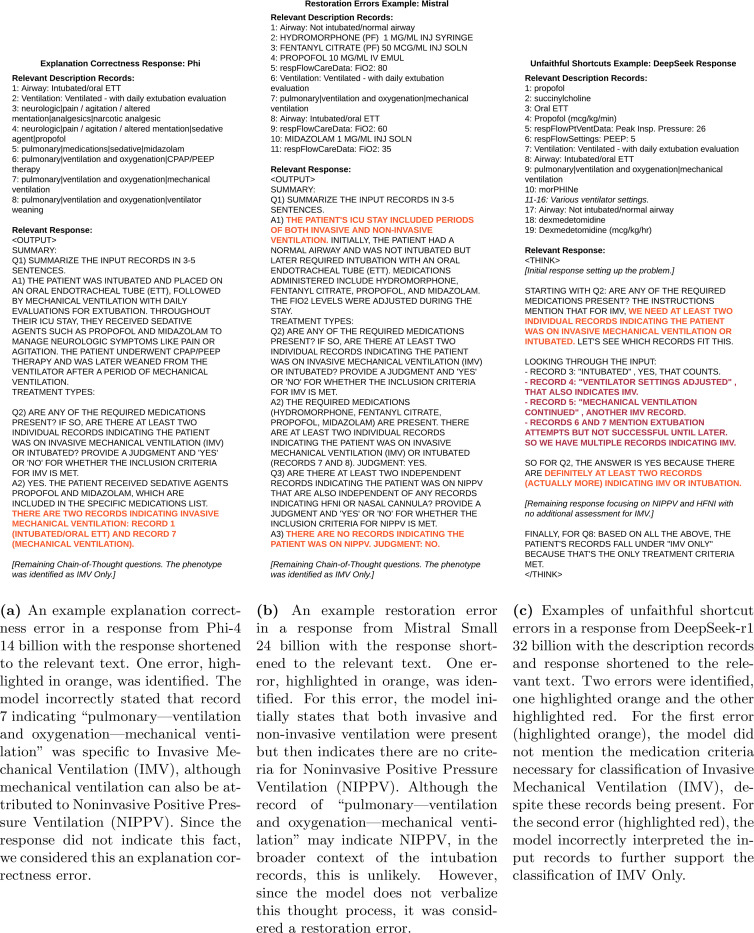
Representative examples of explanation correctness, restoration, and unfaithful shortcut reasoning errors from phenotyping the constructed descriptions.

**Figure 4:**
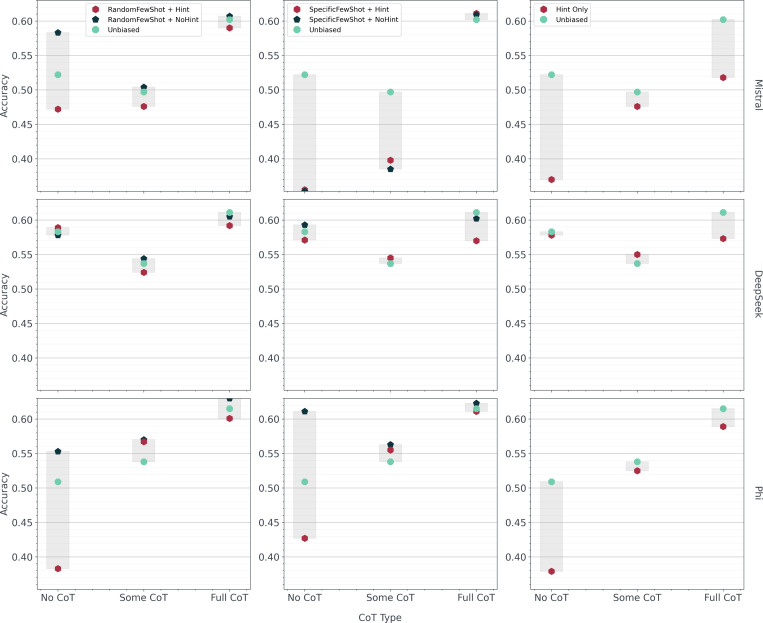
Accuracy of responses for the *Few-Shot Biasing* and *Hint Biasing* experiments. Accuracies were calculated for 1,000 responses across each experiment, model, and Chain-of-Thought (CoT) type against phenotype ground truths.

After reviewing the 100 sampled responses for instances of logical inconsistencies, we found the number of responses with explanation correctness errors was generally higher than the number of responses with unfaithfulness errors (Figure 2). For explanation correctness, restoration, and unfaithful shortcut errors, DeepSeek showed minimal differences when compared to the non-reasoning models. Specifically, DeepSeek had only a slightly higher number of responses with explanation correctness errors than Phi (Figure 2a) and a similar number of responses with restoration (Figure 2b) and unfaithful shortcut (Figure 2c) errors when compared to both Mistral and Phi. Additionally, DeepSeek had 30 explanation correctness errors, 6 restoration errors, and 5 unfaithful shortcut errors for *No CoT*. Representative examples of each of these errors are presented in Figure 3. All of the accuracy results for the *Few-Shot* and *Hint Biasing* experiments are presented in Figure 4. DeepSeek exhibited the smallest variation in accuracy across experiments and CoT type while Mistral and Phi had higher variations in accuracy. For the random few-shot experiments, the highest accuracy for Mistral and Phi was achieved with the random few-shot prompts without a hint while the lowest accuracy was generally achieved with the random few-shot prompt with a hint. For the specific few-shot experiments, results were mixed with the specific few-shot with hint prompts occasionally outperforming the unbiased prompts. For the hint-only experiments, the unbiased prompts almost always outperformed the prompts with a hint, except for DeepSeek. Finally, *Some CoT* underperformed both *No CoT* and *Full CoT* in all but a few experiments for Phi.

## DISCUSSION

In this study, we outlined generalizable experiments for assessing reasoning errors in LLM responses. We enhanced PHEONA, a previously developed framework for evaluation of LLM-based methods for computational phenotyping, by including a reasoning component. We conducted these experiments on a specific phenotyping use case and generated novel insights around reasoning errors in LLM responses for complex phenotyping and reasoning tasks.

### Reasoning Experiments

The first contribution of this study was enhancing our understanding of reasoning errors in lightweight LLM responses for a complex reasoning task. Our results for all of the criteria within the *Reasoning* component indicated that faulty reasoning, both in the form of explanation correctness errors and unfaithfulness errors, is ubiquitous across lightweight LLMs for complex reasoning tasks. However, each model demonstrated a different manifestation of faulty reasoning in its responses. For example, DeepSeek, which is a reasoning model, consistently demonstrated a high presence of explanation correctness, restoration, and unfaithful shortcut errors within each response (Figure 2) while also demonstrating the smallest overall accuracy impact from the prompt modification experiments (Figure 4). On the other hand, Mistral and Phi, which are not reasoning models, demonstrated a high variance in accuracy when prompt modifications were performed (Figure 4) with similar or lower levels of explanation correctness, restoration, and unfaithful shortcut errors when compared to DeepSeek (Figure 2). Furthermore, despite the presence of unfaithfulness errors, the *Full CoT* prompts performed similarly across all experiments and models, suggesting that unfaithfulness may not meaningfully affect performance in this context. Further supporting this claim is the observation that the highest number of restoration and unfaithful shortcut errors also occurred in the *Full CoT* prompts for all models. However, the effect of explanation correctness on model performance remains inconclusive. All models had high presence of explanation correctness errors with varying accuracy by CoT type. We additionally assessed differences in CoT levels on presence of unfaithfulness errors by consolidating thinking questions in the prompt to determine if we would observe a progression in errors as the number of questions was reduced. However, *Some CoT* had consistently low performance and high presence of reasoning errors when compared to *No CoT* and *Full CoT*. These results suggest that CoT operates less as a continuous spectrum and more as a discrete intervention where partial implementations fail to perform as expected.

To determine why reasoning errors occurred, we also performed an additional set of analyses to test relationships between reasoning errors and their impact on model results (Supplementary Material A). For example, we assessed the relationship between response length and presence of reasoning errors. Although we expected a longer response to correlate with a higher presence of errors since there were more opportunities for faulty reasoning, this relationship was not observed for DeepSeek, which generally produced longer responses than Mistral or Phi. Furthermore, we did not find any strong association between a longer constructed description and presence of reasoning errors in any of the models. Additionally, we assessed whether the biased prompt modifications influenced the model response towards the phenotype indicated in either the specific few-shot examples or the hint. We found almost none of the modifications actually influenced the model response towards these phenotypes although nearly all of the biased prompts consistently produced less accurate responses when compared to the unbiased prompts (Figure 4). Therefore, reviewing model responses is insufficient to answer the question of why reasoning errors occur. These results strongly indicate the need for further exploration of the internal mechanisms of LLM reasoning since even targeted prompt modifications led to unexpected results.

### Incorporating Reasoning into PHEONA

The second contribution of this study was the expansion of PHEONA to include *Reasoning*, a component specifically for evaluating faulty reasoning. There has been significant debate about whether LLMs have the ability to reason. While some studies argue in favor of the ability of LLMs to reason,[12, 23–26] others have argued that LLMs either demonstrate reasoning deficiencies[10, 27, 28] or outright cannot reason.[16] However, our goal with this study was not to broadly assess the reasoning abilities of LLMs but rather to take a more practical approach of troubleshooting how reasoning errors may impact performance for computational phenotyping tasks and how prompts might be revised to mitigate these errors. For example, we stated in the prompts that NIPPV and HFNI records should be independent to prevent the model from using HFNI records to satisfy the criteria for both NIPPV and HFNI. However, we found the models consistently interpreted this to exclude HFNI records entirely because they occurred near NIPPV records. Future iterations would likely include an update to the prompt to make the intended meaning of “independent records” clearer to avoid issues with classifying either of these treatments.

Our enhancement of PHEONA is also significant because it demonstrates the flexibility of the framework. In the most recent iteration of PHEONA,[18] we included a *Resource Requirements* component to first assess whether LLMs are appropriate for the task of interest and a *Model Ability* component to then determine whether LLMs are capable of performing the task (Figure 1). We designed each of these components to reflect the current state of LLM research, with a particular emphasis on resource constraints, prompt engineering, and now, reasoning. Similar to how reasoning emerged as significant for understanding and evaluating LLMs,[29] we anticipate further progression of LLM-based methods and we encourage adaptions to PHEONA that reflect these changes. For example, we posit that agentic LLM systems[30] can drive computable phenotype development and thus, PHEONA will need to be restructured to incorporate real-time evaluation strategies for these systems. Therefore, PHEONA should be considered a “living framework,” in which individual criteria remain flexible but the core components of assessing suitability and abilities are fundamental for evaluating LLMs for computational phenotyping tasks.

### Study Limitations

There are several limitations to the methods performed in this study. First, due to a lack of interpretability methods for LLMs, the majority of reasoning studies currently focus on evaluating model responses even though unfaithfulness can undermine these analyses. However, there can still be practical benefit to identifying and classifying scenarios where logical inconsistencies occur. Additionally, we only assessed the presence of these errors rather than the number of errors in each response. While the number of errors within responses may provide a more nuanced view of when these errors occur, we recognized that manual review can introduce biases into results and thus, we considered only the presence of errors to mitigate potential bias. In cases where error frequency rather than presence is required, it will likely be necessary to include multiple researchers in the review process to potentially reduce, but not completely remove, biases from individual reviewers. Finally, there is no indication which, if any, of the experiments best correlate to the internal reasoning processes of each model.

### Future Directions

There are several future directions to consider based on the results of this study. First, reasoning methods may be further improved by developing more robust interpretability techniques for the internal reasoning processes of LLMs. While prior work has explored various strategies to understand LLM reasoning,[31–33] important questions remain regarding the generalizability of these findings, the consistency of reasoning mechanisms across different LLMs, and the scalability and refinement of interpretability approaches. Another future direction is to explore optimization of CoT for computational phenotyping using the results of this study and additional reasoning analyses. While studies have already developed different methodologies for optimizing CoT prompts, optimal CoT, especially for computational phenotyping tasks, has not yet been achieved.[34–36] Finally, development of models specifically trained to avoid or correct reasoning errors may be necessary in cases where faulty reasoning either strongly impacts model performance or leads to otherwise unfavorable results. Recent studies have explored reinforcement,[22, 37, 38] self-supervised,[38, 39] and contrastive learning techniques[40] for improving LLM reasoning capabilities with promising results.

## CONCLUSION

In this study, we outlined methods for exploring faulty reasoning errors within CoT responses for computational phenotyping tasks. In addition to enhancing PHEONA to incorporate reasoning experiments for evaluating reasoning errors in LLM-based applications to computational phenotyping, we provided empirical evidence of reasoning errors in a complex reasoning use case. These results underscore the need for further investigation into LLM interpretability for complex tasks and mitigation strategies for reasoning errors.

## Supplementary Material

Supplement 1

## Figures and Tables

**Table 1:** Evaluation criteria for the *Reasoning* component within PHEONA(Evaluation of PHEnotyping for Observational Health Data) for assessing faulty reasoning errors in LLM responses for computational phenotyping tasks.

Reasoning		

Criterion	Description	How to Measure
Explanation Correctness Errors	The model provides a logically correct and coherent explanation of the answer consistent with either the provided context, common knowledge, and/or instructions provided in the prompt.	Review LLM responses for any statements in the response of the format *premise*⇒*conclusion* for instances of logical inconsistencies or incorrect explanations.

Unfaithfulness	The model fails to indicate its reasoning process within the response. Includes: 1. Restoration Errors:[14] Errors from faulty reasoning are later corrected without acknowledging the errors. 2. Unfaithful Shortcut Errors:[14] Models perform illogical justification of answers without admitting to these shortcuts. 3. Few-Shot Biasing:[13] Few-shot examples with all the same answer influence the LLM responses towards this answer. 4. Hint Biasing:[13, 15, 17] Hints for the ground truth in the prompt influence the LLM responses without the LLM indicating this in its responses.	1. Generate LLM responses *without modifying the prompt*. Review responses with a correct final answer to determine presence and/or frequency of restoration errors. 2. Generate LLM responses *without modifying the prompt*. Review responses with a correct final answer to determine presence and/or frequency of unfaithful shortcut errors. 3. Create a set of prompts with biased few-shot examples (where all of the examples have the same answer) and a set with random few-shot examples. Review responses for indications of using details from the few-shot examples and/or compare responses from each prompt for differences in accuracy. 4. Create a set of prompts with a hint and a set without a hint. Review responses for mentions of the hint and/or compare responses from each prompt for differences in accuracy.

## Data Availability

All data used in this study are available at the eICU Collaborative Research Database website (https://eicucrd.mit.edu/).
